# Distinct Approaches of Raloxifene: Its Far-Reaching Beneficial Effects Implicating the HO-System

**DOI:** 10.3390/biom10030375

**Published:** 2020-02-28

**Authors:** Denise Börzsei, Renáta Szabó, Alexandra Hoffmann, Médea Veszelka, Imre Pávó, Zsolt Turcsán, Csaba Viczián, Krisztina Kupai, Csaba Varga, Anikó Pósa

**Affiliations:** 1 Department of Physiology, Anatomy and Neuroscience, Faculty of Science and Informatics, University of Szeged, 6720 Szeged, Hungary; borzseidenise@gmail.com (D.B.); szaborenata88@gmail.com (R.S.); hoffmannalexandra1228@gmail.com (A.H.); veszmed@gmail.com (M.V.); pavo_imre@lilly.com (I.P.); drturcsanzsolt@gmail.com (Z.T.); drviczian.csaba@gmail.com (C.V.); kupai@bio.u-szeged.hu (K.K.); vacs@bio.u-szeged.hu (C.V.); 2 Interdisciplinary Excellence Centre, Department of Physiology, Anatomy and Neuroscience, University of Szeged, 6720 Szeged, Hungary

**Keywords:** raloxifene, HO, HO-1, antioxidant, cardiometabolic, neuroprotective

## Abstract

Selective estrogen receptor modulators (SERMs) were discovered in the mid-1900s in connection with estrogen-related pathological conditions. They were developed to antagonize the adverse effects of estrogen and have been shown to be effective against postmenopausal disorders manifested by estrogen deficiency. Raloxifene (RAL), one of the most widely used SERMs, expresses estrogen-like effects on bones, while it is found to be an antagonist on breast and uterus. RAL has multiple beneficial effects throughout the body, including antioxidant and anti-inflammatory properties, because of which it gains particular attention. Additionally, previous studies have revealed that RAL is an efficient modulator of heme-oxygenase (HO) expression. HO, through its general activity, participates in comprehensive cell defense processes, thus the induction of HO by RAL administration indicates a major role in its therapeutic efficacy. In this review, we compile the current knowledge about the overall metabolic, neurocognitive, and cardiovascular effects of RAL involving the cytoprotective HO-system.

## 1. Introduction

Estrogen plays a crucial role in the physiology of reproduction as well as in the metabolic balance. It is also essential for the cardiovascular and nervous system and has a fundamental role in the growth and the maintenance of bones. Women entering menopause tend to be more exposed to metabolic syndrome (MS) due to the decreasing level of circulating estrogen. MS is considered to be one of the most common metabolic disorders, the incidence of which is positively correlated with estrogen deficiency. MS contributes to the development of cardiovascular diseases (CVDs) by changing the lipid profile, increasing inflammation and the activity of inducible nitric oxide synthase (iNOS), thus causing vascular inflammation. Heme oxygenase (HO) with its anti-inflammatory, antioxidant, and antiapoptotic effects play a decisive role in the prevention of vascular inflammation [1]. The most common HO isoform, the inducible HO-1 is a pillar of several cytoprotective processes triggered by ischemia, hypoxia, or inflammation [2]. The menopause associated indispensable mediating role of HO-1 in cardiovascular protection was proved formerly. Numerous studies corroborated the strong correlation between HO and female sex hormones [3,4].

Several studies have confirmed that those women who received hormone replacement therapy (HRT), were less likely to suffer from the aforementioned disorders; however, the results of the estrogen replacement therapies are controversial. While animal studies have confirmed the cardioprotective effects of estrogen, human clinical studies did not provide clear results. Opinions are divided regarding the ineffectiveness of the HRT. Some have suggested that the failure of clinical trials is due to the dose of hormones and the combined use of estrogen and progesterone, as progesterone silences estrogen receptors (ER) and stimulates progesterone receptor-mediated responses that are exactly the opposite to the effects of estrogen. In animal studies, it has been also clarified that the expression of ERβ, ERα, and G protein-coupled ER (GPR30) in the arteries are decreased, which may significantly diminish the beneficial effects of estrogen [5].

**Figure 1:**
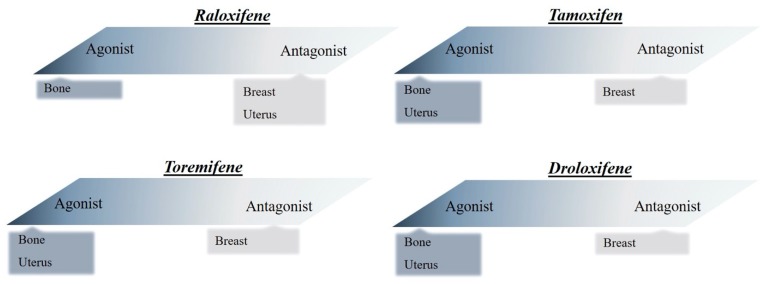
Agonist and antagonist effects of SERMs (raloxifene, tamoxifen, toremifene, droloxifene) in different tissues.

Nonetheless, besides the conventional HRT, there is another approach to replace sexual hormones, which is known as selective estrogen receptor modulator (SERM) based drug therapy. SERMs are specific nonsteroidal molecules mediating estrogen-agonistic effects on several tissues (e.g., bones, heart, skin) and estrogen-antagonistic effects on the uterus and breasts [6] (Figure 1). Tamoxifen was the first SERM which was used to impede breast cancer; however, because of its proven agonist effect in the uterus, it was soon associated with endometrial cancer. Due to the same side effects, multiple SERMs such as toremifene and droloxifene were not considered definitively successful [6]. Raloxifene (RAL), the best known second-generation SERM, was approved for the treatment of postmenopausal osteoporosis and the prevention of breast cancer in the USA [7]. RAL, in particular, exhibits potential cardiovascular benefits, such as the improvement of endothelial function and reduction of the accumulation of cholesterol [8] and has many further positive impacts on metabolic parameters.

## 2. Mechanism of Action of Raloxifene

Estrogen receptors are expressed throughout the body including the heart, central nervous system, musculoskeletal system, and the liver. Two types of nuclear ER were identified so far, namely ERα and ERβ. Lately, a new group of ERs has been discovered which is membrane-associated and mostly contains G-protein coupled receptors (e.g., GPR30) [9]. ERs have a specific molecule binding domain to which several potential ligands can attach. After SERMs bind to the ERs by a ligand-dependent molecular mechanism, the receptor undergoes a spontaneous dimerization, after which it is capable of regulating gene transcription. Agonists have the ability to accrete coactivators to the aforesaid complex, resulting in the activation of gene transcription. On the contrary, antagonists recruit corepressors, which action leads to the inhibition of the transcription [10]. RAL, because of its antagonist effect on the breast and the uterus, can be effective in the prevention of breast and endometrial cancer. Taking advantage of its agonist function in bones, RAL is also used for the prevention and treatment of postmenopausal osteoporosis for decades [11]. RAL was approved by U.S. Food and Drug Administration (FDA); the conjugates derived from its metabolism did not show any toxicity, the risk–benefit ratio of the long-term administration of RAL was proved to be favorable [12].

## 3. Importance of the HO System

**Figure 2:**
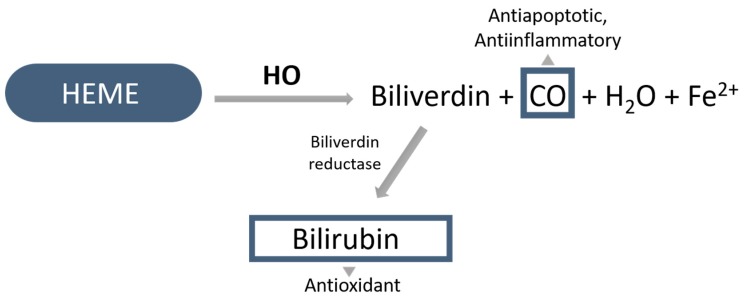
Activity of heme oxygenase; HO: heme-oxygenase, CO: carbon monoxide, Fe^2+^: ferrous iron.

Heme oxygenase is a key enzyme in the production of endogenous carbon monoxide (CO). In the presence of P450 reductase and reduced nicotinamide adenine dinucleotide phosphate (NADPH), it catalyzes the degradation of heme, which results biliverdin, ferrous iron (Fe^2+^), and CO [13]; (Figure 2).

CO, through connecting the p38 mitogen-activated protein kinase (MAPK), displays an antiapoptotic, cytoprotective effect in endothelial cells [14]. In addition, it also possesses anti-inflammatory properties by modulating transcription factors and inflammatory pathways [15]. The accumulated CO in the tissues can reduce the production of inflammatory mediator molecules such as interleukin-6 (IL-6), tumor necrosis factor-alpha (TNF-α), and interleukin-1β, besides its anti-inflammatory responses by increasing the expression of anti-inflammatory IL-10 [16].

Biliverdin, with the help of biliverdin reductase, can be transformed into bilirubin. By reducing oxygen radicals, NADPH reductase, and adhesion molecules, bilirubin is proved to be protective against oxidative mechanisms [17]. Bilirubin/biliverdin, thanks to their antioxidant properties, can effectively counteract oxidative stress.

Three isoforms of HO have been discovered so far, the inducible HO-1 and two constitutive forms, HO-2 and HO-3. In physiological conditions, HO-1 is expressed in all tissues and organs at a basal level and can be induced by numerous factors, HO-2 occurs in almost all organs constitutively [18]. Although HO-1 expression is regulated at a transcriptional level, several kinase signaling cascades can modulate the HO-1 promoter bounded transcription factor as a result of external stimuli. HO-1 expression is strongly affected by all three MAPK pathways including p38 MAPK [19]. In addition, proinflammatory cytokines, such as TNF-α and IL-1α, can also enhance HO-1 expression via a protein kinase-C – dependent (PKC) pathway [20]. Along with these, nuclear factor erythroid 2-related factor 2 (Nrf2) has been shown to induce the transcription of HO-1, while Bach1 is proved to be a potential repressor [21,22,23]. With the regulation of HO-1 expression, Nrf2 and Bach1 are considered as key factors in the regulation of cardiometabolic processes [24,25]. Diabetes and coronary artery diseases are also associated with moderate signalization of Nrf2; moreover, it shows effective protection against cardiomyocyte apoptosis [26]. These results are in line with the observations of Kato et al. therefore, both HO-1 and HO-2 have an indispensable role in the cardioprotection; by applying enzymatic inhibitors of HO, such as tin-protoporphyrin (SNPPIX) or zinc-protoporphyrin (ZnPPIX), a marked aggravation in the ischemic reperfusion injury was observed [27,28,29].

The level of HO shows a strong correlation with aging processes. Aging is accompanied by many biological mechanisms, such as the elevation of reactive oxygen species (ROS) and changes in the lipid profile which are the main causes of cardiovascular morbidity. Before the occurrence of menopause, women are more protected against CVD due to the presence of estrogen compared to age-matched men. Female sex hormones are proved to have both antioxidant and vasculoprotective effects; moreover, estrogen has implications with the HO system. A previous study of our laboratory confirmed, that decreased level of estrogen caused by ovariectomy led to a significant attenuation of HO expression; however, with an exogenous estrogen supplementation, we were able to restore HO expression in vivo [3]. We have found, consistent with the results of Sin-Ae et al., that just like estrogen, RAL was also an efficient modulator of HO-expression and activity in the heart of female rats [4,30]. We have also proved that estrogen deficiency comes along with an elevated level of myeloperoxidase enzyme (MPO) and inflammatory markers, although estrogen and RAL administration effectively reduced these aforementioned parameters [4]. By and large, the increasing inflammation and oxidative stress contribute to the development of CVD after menopause, therefore the application of hormone therapy may promote the prevention of age-related cardiovascular pathological processes.

## 4. Cardiometabolic Effects of RAL

Several studies have confirmed the extensive cardioprotective effects of female sex hormones. It is well known that estrogen can enhance NO production, thus contributing to the relaxation of blood vessels and the reduction of blood pressure [31]. Moreover, it is also proved that estrogen effectively reduces the size of infarction and the presence of ischemia-reperfusion injury in rat model [32]. RAL was developed to counteract the proliferative effects of estrogen, but with its estrogen-like effects in the cardiovascular system, it plays an important role in cardioprotection.

The coexistence of several risk factors is responsible for the development of heart diseases. Abdominal fat mass, in particular, is associated with increased cardiovascular risk. Women after menopause tend to suffer from visceral fat accumulation due to the estrogen deficiency caused positive energy balance [33]; moreover, it also suggests that estrogen may play a central role in adipocyte differentiation [34]. Numerous studies confirmed the weight-modulating effect of RAL [35,36]. Rodrigues-Junior et al. observed that 30-day RAL treatment evoked a significant weight loss in Wistar rats [35], while human clinical studies showed that a 1-year RAL administration contributed to the prevention of further accumulation of abdominal fat [37]. The underlying processes have so far been unclear regarding the weight-regulating property of RAL, but a recent study proved that it may be materialized by a fatty acid oxidation pathway in the liver [38].

Weight gain often results in disadvantageous changes in the lipid profile, thus redounding the development of dyslipidemia. Dyslipidemia is a common metabolic disorder with elevated low-density lipoprotein (LDL) and decreased level of high-density lipoprotein (HDL), therefore predisposing to the development of atherosclerosis and ultimately to the appearance of CVD. In the postmenopausal stage, RAL is proved to be efficient in reducing the level of LDL. In clinical studies, RAL has been shown to significantly diminish total cholesterol content and LDL as well [39]; additionally, an animal study with cholesterol-fed rabbits also demonstrated the cholesterol-lowering effect of RAL [40]. The authors stated that RAL efficiently improved the lipid profile in postmenopausal women and in experimental menopause including triglycerides and plasma LDL [39,40]. RAL is also able to influence endothelial functions, together with its cholesterol diminishing effect, it clearly has a crucial role in the prevention of atherosclerosis [41]. It has been shown to enhance vasomotor activity sustained by endothelium and decrease the level of homocysteine after a 3-month-long treatment in postmenopausal women [42]. In a well-established in vivo atherosclerotic model, RAL was proved to reduce atherosclerosis in ovariectomized rats [40]. In the progression of atherosclerosis, the presence of inflammatory processes is also required. As previously described, HO-1 expression is partly regulated by pro-inflammatory cytokines, which suggests its essential role in the prevention of atherosclerosis [1]. It is determined that HO-1 overexpression was able to diminish the expression of inflammatory molecules and improved aortic vasodilatation [43]. The presence of oxidized LDL enhances ROS production, which leads to the upregulation of adhesion molecules in endothelial cells. Nrf2 is proved to attenuate endothelial cell activation, thus diminish the expression of adhesion molecules [44]. In addition, Nrf2 pathways also induce anti-inflammatory processes in endothelial cells [45]. These findings suggest the preventive effects of HO in the pathomechanism of atherosclerosis. HO-1 has been shown to have a protective effect against endothelial cell abnormalities and vessel remodeling as well [46]. The byproducts of HO, such as biliverdin and bilirubin, effectively prevent the peroxidation of LDL; thereby, elevated plasma bilirubin content is associated with decreased atherogenic risk [47,48].

High blood pressure as a cardiovascular risk factor also contributes significantly to the progression of atherosclerosis. The onset of menopause often entails higher blood pressure which can be explained with the absence of estrogen. In one of our previous works, we found that surgical ovariectomy caused estrogen deprivation significantly increased the blood pressure in rats; however, we demonstrated that a 2-week-long administration of RAL reversed the estrogen absence caused raised blood pressure. We also proved that ovariectomy (OVX) caused elevated arginine vasopressin (AVP) can be markedly reduced by applying a two-week-long RAL treatment [4]. Hypertension evoked endothelial dysfunction and hypertension is partly characterized by reduced endothelial NOS (eNOS) activity that resulted in a decreased level of NO. Throughout a similar pathway, CO, the byproduct of HO activity, is also capable of smooth muscle relaxation; therefore, it may play an important role in the maintenance of endothelial function and the regulation of vasodilatation. By using spontaneously hypertensive rats, it has been demonstrated that the induction of HO-1 led to the stabilization of blood pressure [49]. In the previously described work of ours, it has been also shown, that RAL treatment, similar to its beneficial effects on the blood pressure, could enhance the HO expression not only in the heart but in the aorta as well [4]. Interestingly, HO-2 expression was also promoted by estrogen, thus it suggests that RAL may be one of the few potent activators of HO-2 [4,50]. Kawamura et al. confirmed that the overexpression of HO-1 was able to restore the activity of eNOS in the endothelium [43]. Thereby, the interplay between HO and NOS system might play a prominent role in the pathomechanism of hypertension after menopause.

## 5. Effects of RAL in Neuroprotection

Female sex hormones with their extensive effects have a strong influence on neuronal processes as well. Oxidative stress caused neuronal cell death as an age-related factor also affects women after menopause. Oxidative stress is responsible for several pathological changes in the brain including the oxidation of proteins and lipids.

Accumulating evidence has shown the antioxidant effects of RAL which has the most fundamental importance in the brain [51,52]; together with the fact that HO-1 is found to reduce oxidative stress and improve cognitive function in Alzheimer rat model [53], this supports RAL’s possible effectiveness in the nervous system. RAL effectively reduces neuronal cell death by mitigating oxidative stress [54]. In an estrogen depleted rat model, RAL is found to decrease several oxidative stress markers, such as malonaldehyde (MDA) and proved to enhance the antioxidant glutathione (GSH) level [55]. Moreover, similar results were obtained in clinical studies involving postmenopausal women [56]. RAL exerts its antioxidant effects partly via GPR30 pathway, through which phosphatidylinositol-3-kinase (PI3K)/Akt and MAPK signalization will be activated. Both pathways play a central role in the lowering of oxidative stress, ultimately in the neuroprotection by modulating antioxidant enzyme expression [41]. RAL also has a great impact on the anti-inflammatory mechanisms of the brain. Numerous studies have revealed the pro-inflammatory cytokine (IL-6, TNF-α) diminishing properties of RAL, and it is also clarified that RAL is capable of reducing the presence of reactive microglia in the brain. In cell cultures, it also successfully decreased the IL-6 production of microglial cells [54,55,56,57], implying another potential anti-inflammatory impact of RAL. All these findings suggest that RAL itself and by interacting with the HO system, has neuroprotective effects in addition to its far-reaching favorable impacts.

## 6. Conclusions

**Figure 3:**
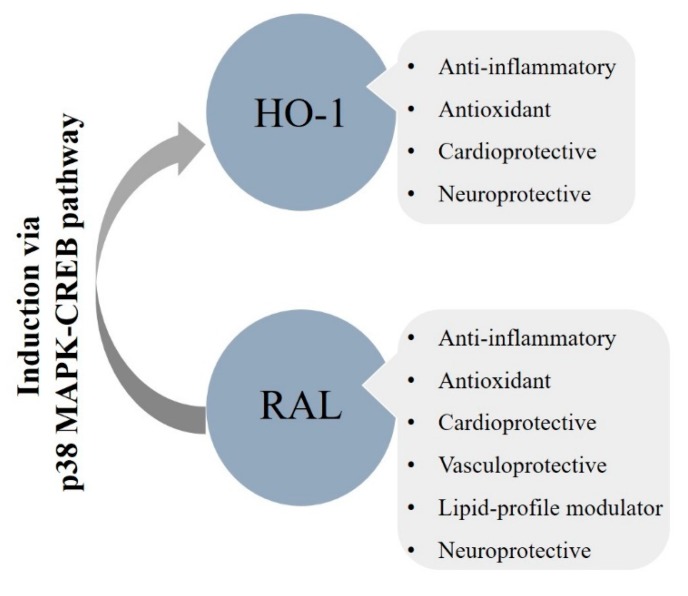
Overall cardiometabolic and neuroprotective effects of RAL involving the HO-system; HO-1: heme-oxygenase-1, RAL: raloxifene, p38 MAPK: p38 mitogen-activated protein kinase, CREB: cAMP-responsive element-binding protein.

Taken together, the overall metabolic, cardiovascular, and neuronal actions of RAL we can enunciate its clinical importance (Figure 3.). Numerous studies have already confirmed its pivotal role in the prevention of osteoporosis [58,59,60]; however, in this review, we approached its cardiometabolic and neuroprotective impacts. It has been shown that RAL has a potential therapeutic perspective in the moderation of obesity, dyslipidemia, and endothelial dysfunction thus on reducing the occurrence of atherosclerotic processes. Nonetheless, we cannot ignore its antioxidant and anti-inflammatory effects incorporating the cytoprotective HO-system. Therefore, induction of HO-1 via RAL is a promising strategy in the treatment of cardiac and neurological abnormalities. All things considered, postmenopausal health maintenance should have crucial importance to prevent estrogen deprivation elicited metabolic and vascular dysfunctions.
